# Targeted muscle reinnervation in upper extremity amputations

**DOI:** 10.1007/s00590-023-03736-2

**Published:** 2023-10-09

**Authors:** Elliot L. H. Le, Matthew L. Iorio, Mark A. Greyson

**Affiliations:** Division of Plastic and Reconstructive Surgery, University of Colorado Anschutz Medical Center, 12631 East 17Th Ave, Room 6111, Aurora, CO 80045 USA

**Keywords:** Targeted muscle reinnervation, Upper extremity amputation, Peripheral nerve surgery

## Abstract

Targeted muscle reinnervation (TMR) is a relatively recent surgical innovation that involves the coaptation of major peripheral nerves to a recipient motor branch that innervates an expendable muscle target. The original indication for TMR was augmentation and optimization of myoelectric signals in the amputated limb for use of myoelectric prosthetics. Incidentally, surgeons and patients discovered that the technique also could treat and prevent phantom and residual limb pain. TMR is performed at the time of amputation or delayed any time after the amputation, and TMR can also be performed at any level of amputation. In the upper extremity, studies have detailed the various techniques and coaptations possible at each amputation level to create intuitive myoelectric signals and treat neurogenic pain. Treatment of peripheral nerves in the amputee with TMR should be a consideration for all patients with major upper extremity amputations, especially at large institutions able to support multidisciplinary limb salvage teams. This review article summarizes the current literature and authors’ techniques and recommendations surrounding TMR in the upper extremity amputee including techniques relevant to each level of upper extremity amputation.

## Introduction

Techniques in major limb amputations have remained largely unchanged in contrast to the rapid refinements in surgical technique across a variety of surgical procedures. Patient-centric outcomes such as pain and the ability to wear and control a prosthetic have been static. However, over the past twenty years, and through the pioneering work of Drs. Paul Cederna and Gregory Dumanian, two surgical techniques have emerged which show great promise in their potential to mitigate neuroma related pain and phantom pain as well as improved control of prostheses. These techniques are targeted muscle reinnervation (TMR) and Regenerative Peripheral Nerve Interfaces (RPNI). TMR involves the end-to-end coaptation of major peripheral nerves to recipient motor nerves in an amputated limb. It was first described by Dr. Dumanian in 2002 in a patient with bilateral shoulder disarticulations in an effort to augment myoelectric signals for improved myoelectric prosthetic control [1]. Serendipitously, this technique has been shown to also significantly ameliorate phantom and residual limb pain, by giving the sensory and mixed nerves “somewhere to go, and something to do [2].”

Commensurate with the developments in surgical technique, advancements in upper limb prosthetics have evolved in recent years. Myoelectric prosthetics use myoelectric signals from the muscles in the residual limb, either through surface or implantable electrodes, and a neural processing system to generate complex movements in a prosthetic. TMR has the potential to augment myoelectric signal and through advanced processing software and create intuitive signals to increase the degree of control and dexterity of the prosthetic. TMR has been performed in patients in whom an amputation has been indicated for a wide variety of reasons, including traumatic, peripheral vascular, diabetic, and oncologic etiologies [1, 3]. Over the past several years, there has been significant growth in the number of publications related to TMR, as well the number of centers now offering this procedure as an adjunct to both upper and lower extremity amputations.

The purpose of this review article is to describe techniques in TMR for upper extremity amputations, as well as provide a literature review on considerations and outcomes of this surgical innovation.

## Preoperative considerations

TMR is an option for any patient with a major upper extremity amputation proximal to the metacarpal-phalangeal joint. Nearly two thirds of trauma-related amputations are of the upper extremity [4]. Amputations are also be performed for patients with unsalvageable burn injuries, dysvascular upper extremities, and oncologic etiologies. Preoperative evaluation includes indication for amputation, indication for TMR, and physical examination of residual motor and sensory function. With regard to amputation level, keeping supple wrist motion including flexion, extension, and supination and pronation is ideal. If a transradial amputation is necessary, targeting a level 5 cm proximal to the radial styloid allows enough room for prosthetic componentry while still maintaining pronation and supination.

The indication for amputation is important to consider as this determines the available options for TMR. Patients undergoing amputation for dysvascular limbs, for example, will require a thorough workup of their vascular anatomy and capacity for wound healing. The level of vascular compromise could be more proximal than the zone of necrosis or gangrene. In the oncologic population, a thorough history of neoadjuvant treatment such as chemotherapy and radiation will also compromise wound healing and may extend the zone of injury beyond what is easily visible. Similarly, patients with crush and avulsion injuries can also result in proximal nerve and vascular injuries, again extending the zone of injury. A thorough physical examination of voluntary contractions of muscles proximal to the injury to identify possible targets for TMR. An electromyography test should be performed preoperatively in the appropriate setting to rule out brachial plexopathies or nerve injuries proximal to the amputation [1, 5, 6]. Patients with compromised wound healing capacity and those without any viable muscle targets will be poor TMR candidates.

There are two main indications for TMR in the upper extremity—(1) to improve prosthetic function through augmentation and optimization of myoelectric signals and (2) to treat phantom and residual limb pain. Either indication can be addressed in an acute or delayed fashion. Acute TMR is performed when patients have a planned amputation either at the time of amputation or within a few weeks [1]. The patient must be able to tolerate a prolonged anesthetic time. These patients will benefit from initiating the reinnervation timeline as quickly as possible to expedite the myoelectric signal development and prevent establishment of chronic pain pathways [7]. These pain pathways lead to phantom limb pain or the sensation of the missing limb following amputation [8, 9]. In a delayed TMR, patients’ preoperative prosthetic function, residual limb pain areas, and phantom limb pain areas are carefully assessed prior to reinnervation to identify the neurogenic nerves to be addressed. In summary, the nature of the amputation and the timing of TMR must all be accounted for in the preoperative plan.

## Surgical technique

### General principles

At its core, TMR is an end-to-end nerve transfer that requires a donor nerve, and a recipient nerve that innervates an expendable muscle target. Therefore, the most distal level at which this procedure may be performed is approximately at the mid metacarpal level, at the level of innervation of the intrinsic muscles of the hand. Digital nerves distal to the intrinsic muscle branches may be addressed through traction neurectomy or RPNI. In the latter, a muscle graft is wrapped around the cut nerve end [10].

The donor nerve in TMR may be any sensory, motor, or mixed motorsensory nerve. The recipient nerve is a motor nerve, which can be identified with a intraoperative peripheral nerve stimulator. The muscle target should be vascularized, but denervated to prevent cross-talk and ensure signals to the target are from the donor nerve only.

The surgery is performed with the patient under general anesthesia without muscle relaxation. An upper extremity regional block may be performed, as this will not impact identification of target nerves using the nerve stimulator. The patient is placed in a supine position for amputations performed at or distal to the humerus and in the lateral decubitus positioning for shoulder disarticulation [5, 6, 11, 12]. Sometimes it is necessary to turn a patient prone for the posterior approach in transhumeral amputees. Although this can be performed through a single anterior incision if performed secondarily or concurrently to the amputation, through volar and dorsal fishmouth incisions [13]. An upper arm tourniquet may be used in amputations distal to the elbow; however, reliable nerve stimulation is only possible within 30 min of tourniquet inflation due to ischemic palsy [14]. Thus, we recommend prudent use of the tourniquet by inflating the tourniquet in multiple short periods to first identify donor and recipient nerves visually. Then deflating the tourniquet to assess for hemostasis and verify motor targets with the stimulator. Finally reinflating the tourniquet to complete the coaptations.

There are no standard incisions for TMR procedures at any of the described amputation levels, as it is paramount to remember that this is not a prescriptive operation. Generally, patients undergoing TMR and amputation simultaneously will have fishmouth flaps and we favor extending those incisions proximally to facilitate exposure of nerves and targets. In a delayed TMR, surgery can be performed through single or multiple incisions proximal to the zone of injury. Full thickness flaps should be elevated to expose the underlying muscle and nerves. Further details on the design of incisions and identification of donor and recipient nerves for each level of amputation will be described in subsequent sections.

Donor nerves are often found as neuromas on the distal limb stump. In the case of TMR for myoelectric prosthetic control when surface electrodes are used, donor nerves are also deemed not useful if their distal target is too deep to produce a meaningful cutaneous myoelectric signal. Recipient muscle targets should be healthy, superficial, and expendable. Loss of the target muscle should have inconsequential effects on function or be part of a redundant function [11]. We recommend also being judicious with regards to denervation of muscle at the amputation stump, as to prevent atrophy and loss of soft tissue bulk at the stump itself. The depth of the muscle target is not a factor when TMR is performed for neuroma or phantom pain only.

After identifying the donor and recipient nerves, the nerves must be transected. The donor nerve is transected as distal as possible while the recipient nerve is transected as proximal as possible to help ensure a tension-free coaptation. Transection is made sharply with a scalpel on a firm surface such as a sterile tongue depressor, or with a neurotome, and trimmed as necessary until healthy, blossoming, and bleeding fascicles are identified. It is unnecessary to excise the remaining distal end of the donor nerve and its neuroma, although they may be removed if bulky. The donor nerve is then coapted to the recipient motor nerve with 8–0 or 9–0 nylon interrupted sutures in the epineurium of both nerve ends under the microscope or loupe magnification. Our preference is to place the minimum number of sutures to ensure tension-free approximation of the epineurium followed by fibrin glue. Placing more than 3–6 sutures can damage fascicles, increase scarring, and lead to suboptimal reinnervation [15, 16]. In cases of diameter mismatch, the coaptation can be wrapped with a nerve wrap or surrounded by denervated but vascularized muscle to prevent axonal escape [1, 13, 17].

Alternatives to suture repair, which can be technically challenging, time intensive, and prone to iatrogenic nerve injury, include bioabsorbable glues [18, 19]. A recent systematic review of both animal and human studies found that off-label use of fibrin glue alone for peripheral nerve repair demonstrated similar nerve regeneration potential to suture repairs [19]. Suture-less repair saves operative time, but may result in a higher incidence of dehiscence, and therefore TMR failure [19, 20]. It is our preference to combine fibrin glue with a few approximating sutures, which facilitates adequate repair alignment and strength [21].

After all coaptations are complete, we ensure each individual TMR sites are isolated from each other physically by burying coaptations in muscle as needed, and bathing the coaptation with fibrin glue sealant. Alternatively, other techniques described include interposing adipofascial flaps or synthetic grafts between coaptations to minimize cross-talk of myoelectric signals [5, 11].

Closure is completed with muscle myodesis to improve coverage of the residual limb in transradial and transhumeral amputations and layered closure of the skin flaps. It can be helpful to mark the recipient muscle bellies of each TMR performed on the closed limb and take a picture for the patient’s records as a map for eventual electrode placement of the prosthetic [11].

TMR technical details vary based on the level of amputation. These techniques, preferences and considerations are detailed below in each subcategory.

### Shoulder disarticulation

The first described TMR was performed in a patient with bilateral shoulder disarticulations in 2002. TMR at this level of amputation is much less predictable and coaptation combinations vary more than other levels [22]. Major peripheral donor nerves include the musculocutaneous, median, ulnar and radial nerves. Target muscles of interest are in the lateral chest and back including the latissimus dorsi, pectoralis major sternal and clavicular heads, pectoralis minor, and serratus anterior (Table 1).

**Table 1:** Summary of the surgical approach, donor nerves to be addressed, available recipient nerves and the ideal coaptations to facilitate myoelectric prosthetic control in patients with a shoulder disarticulation

Surgical approach	Donor nerve	Recipient nerve options	Ideal combination for myoelectric prosthetic control
Incision 2–3 cm below inferior border of clavicle exposing pectoralis major and brachial plexus	Musculocutaneous Median Ulnar Radial	Pectoralis major clavicular head, Pectoralis major sternal head – split branches, Thoracodorsal Long thoracic, Pectoralis minor	Musculocutaneous → Pectoralis major clavicular head Median → Pectoralis major sternal head Ulnar → Pectoralis major sternal head Radial → Thoracodorsal

**Fig. 1:**
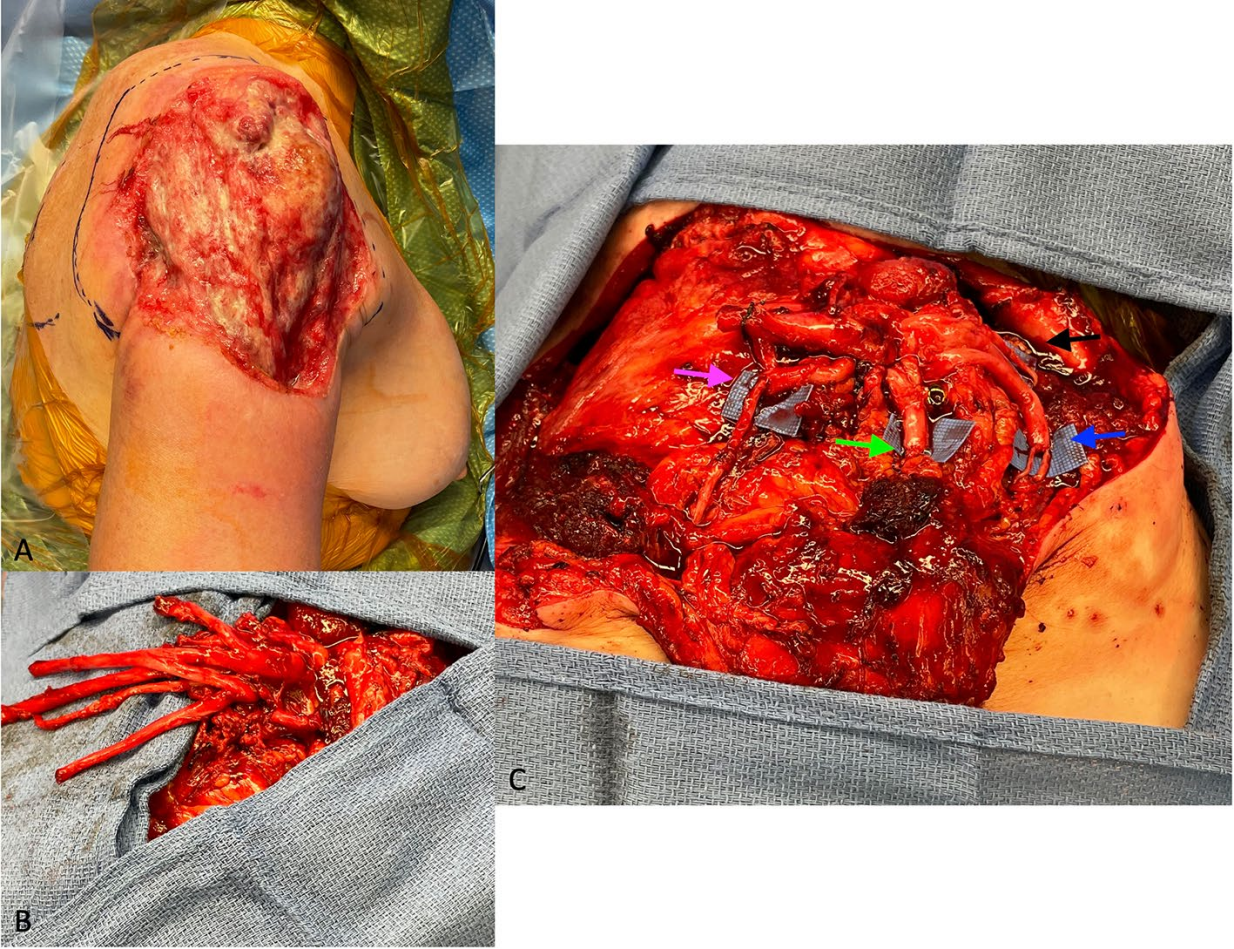
**a**–**c**. 67-year-old woman with a 20-year history of locally invasive basal cell carcinoma on the right shoulder requiring a shoulder disarticulation with TMR (1**a**). The branches of the brachial plexus were dissected (1**b**) and then coapted to the motor branches in the pectoralis major (PMaj) and thoracodorsal nerves. (1**c**) The musculocutaneous nerve was transferred to the clavicular head branch of PMaj (black arrow), median nerve to the sternal head branch of PMaj (blue arrow), ulnar nerve to an additional sternal head branch of PMaj (green arrow), and the radial nerve to the thoracodorsal nerve (pink arrow) (color figure online)

The incision is designed 2–3 cm below the inferior border of the clavicle from the medial chest to the distal axilla to widely expose the pectoralis major and brachial plexus. The sternal and clavicular heads of the pectoralis major are identified, and a space between the two is developed bluntly. Deep to both muscle heads will be the thoracoacromial pedicle and the three motor nerves to the pectoralis major sternal and clavicular heads—superior pectoral nerve, middle pectoral nerve, and inferior pectoral nerve. All three nerves must be identified for denervation purposes and TMR preparation. The sternal and clavicular heads may be split and either an adipofascial flap or synthetic graft should interpose the two to minimize cross-talk. The brachial plexus is located in the fat in between the two pectoralis major heads and deep to pectoralis minor. The pectoralis minor can be divided to facilitate access to the plexus. Fortunately, identification of each major peripheral nerve is unnecessary and coaptation patterns are based on the position of the individual donor and recipient nerves. A case example of acute TMR in shoulder disarticulation is described in Fig. 1.

### Transhumeral

The transhumeral amputee has three major peripheral nerves to consider—median, ulnar, and radial nerves. Transfers at this level are limited in variations and thus widely standardized as the combinations address pain and myoelectric prosthetic control well (Table 2).

**Table 2:** Summary of the surgical approach, donor nerves to be addressed, available recipient nerves and the ideal coaptations to facilitate myoelectric prosthetic control in patients with a transhumeral amputation

Surgical approach	Donor nerve	Recipient nerve options	Ideal combination for myoelectric prosthetic control
Anterior incision over the biceps raphe for median and ulnar nerve coaptations Posterior incision over the triceps raphe for radial nerve coaptation	Median Ulnar Radial	Anterior Biceps brachii short head Biceps brachii long head brachialis Posterior Triceps brachii lateral head Triceps brachii long head Triceps brachii medial head	Median → Biceps brachii short head Ulnar → Brachialis Radial → Triceps brachii lateral head

The median and ulnar nerve coaptations are performed through an anterior incision. The incision is marked over the raphe between the two heads of biceps brachii. Blunt dissection between both heads of the muscle will reveal the musculocutaneous nerve, which has three motor branches—one to each biceps head proximally and the brachialis branch distally. After identification of the recipient nerves, the short head of biceps (medial muscle belly) is retracted laterally to identify the median nerve adjacent to the brachial artery and the ulnar nerve deep to both structures. Both nerves are transected distally and tunneled deep to the short head of biceps into the inter-biceps space where the coaptation is performed to the branches of the short head of biceps and brachialis, respectively.

The radial nerve TMR is performed through a posterior incision along the raphe between the long and lateral heads of triceps brachii. Cranial retraction of the deltoid’s caudal border will reveal the space between the lateral and long heads of triceps brachii. The radial nerve will be found anterior to the two heads with motor branches entering the lateral head and the coaptation performed to the motor branch of lateral head of the triceps brachii. Although described with two approaches here, the surgery has been done through a single incision [13].

### Transradial

**Fig. 2:**
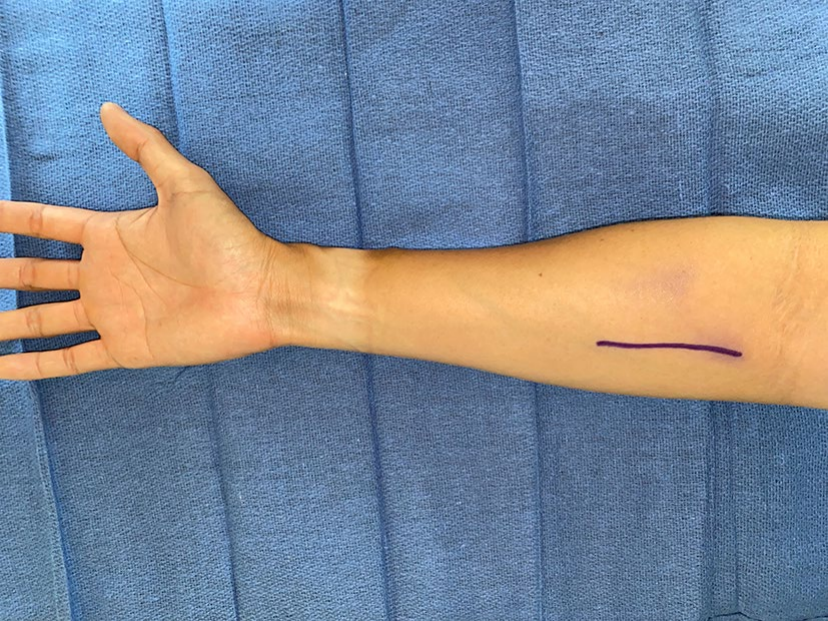
The standard approach for TMR in a transradial amputation is a volar proximal forearm incision that favors the ulnar side to expose the median nerve and ulnar nerves and their targets

Transradial amputations are the second most common upper extremity amputation following partial hand amputations [23]. TMR at this level is often done through a single volar incision in the proximal forearm separate from the amputation stump, especially in the case of delayed TMR (Fig. 2) [6]. In cases of acute TMR in transradial amputations, it is possible to perform the ulnar and median nerve transfers through the traditional fishmouth incisions, but the transfers are quite proximal in the forearm (Table 3) [11, 12].

**Table 3:** Summary of the surgical approach, donor nerves to be addressed, available recipient nerves and the ideal coaptations to facilitate myoelectric prosthetic control in patients with a transradial amputation

Surgical approach	Donor nerve	Recipient nerve options	Ideal combination for myoelectric prosthetic control
Volar proximal forearm incision ulnar to the brachial artery to expose the median and ulnar nerves	Median Ulnar	Palmaris longus Flexor carpi radialis Flexor pollicis longus Flexor digitorum profundus Flexor digitorum superficialis Extensor carpi radialis longus Extensor carpi radialis brevis Flexor carpi ulnaris – two heads Brachioradialis	Median → Flexor digitorum superficialis Ulnar → Flexor carpi ulnaris

Described targets for TMR of the median nerve include palmaris longus (PL), flexor carpi radialis (FCR), flexor digitorum superficialis (FDS), and flexor digitorum profundus (FDP) [6, 11, 12]. Described targets for TMR of the ulnar nerve include flexor carpi ulnaris (FCU) and flexor pollicis longus (FPL) [6, 11, 12]. We prefer identifying all the proximal branches of the median nerve proper and the AIN first. The FDS branch can be identified near the level of the anterior interosseous nerve (AIN) and is transected near its origin on the median nerve. The median nerve is then transected distal to the AIN and FDS branch to preserve proximal motor branches and the two ends are coapted. The same steps can be repeated for the various other alternatives. The ulnar nerve TMR is simpler as the dissection is performed at the interval of both heads of FCU. The FCU branches of either head can be used for TMR. The branch is transected just distal to its takeoff and the ulnar nerve is transected distal to both branches of each FCU head. The two ends are then coapted. These coaptations can be performed through a single volar forearm incision. Major sensory nerves such as the medial antebrachial cutaneous nerve (MABC) and lateral antebrachial cutaneous nerve (LABC) in the forearm can become sources of pain. TMR to available muscle targets can be performed or the sensory nerve buried into muscle bellies or the bone [1, 11, 24].

### Partial hand amputations

Digit and partial hand amputations distal to the wrist are the most common traumatic amputation worldwide [23]. These amputations vary from single digits to transmetacarpal to transcarpal amputations. TMR in the distal wrist and hand will mostly address phantom and residual limb pain. Coaptations will be highly variable and dependent on level of amputation, available recipient nerves, and amputated donor nerves. Depending on the level of the amputation, the individual digital nerves distally or the median nerve, ulnar nerve, and the radial sensory proximally need to be addressed. Intrinsic muscles such as the seven interossei, four lumbricals, hypothenar and thenar muscles serve as viable targets (Table 4).

**Table 4:** Summary of donor nerves to be addressed and available recipient nerves in patients with a partial hand amputation of variable levels including transdigital, transmetacarpal, and transcarpal amputations

Donor nerve	Recipient nerve options
Median Ulnar Radial sensory Digital nerves	Four dorsal interossei Three volar interossei Four lumbrical Deep motor branch of ulnar Recurrent motor branch of median Pronator quadratus

**Fig. 3:**
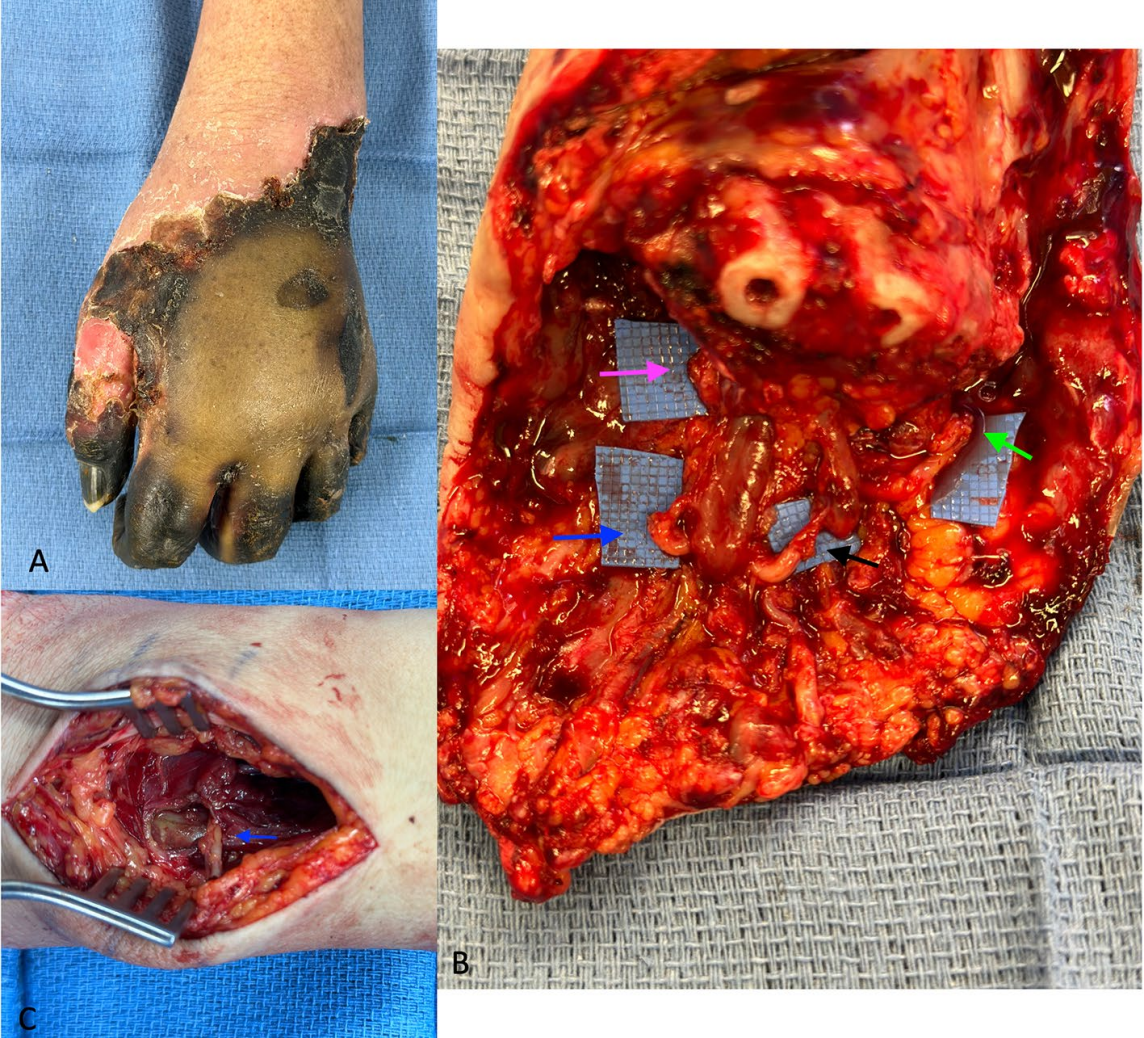
**A**–**C**. 44-year-old woman with a history of septic shock complicated by dry gangrene of the left hand following vasopressor therapy requiring a partial hand amputation with TMR (3**a**). The digital nerves were identified and transected proximal to distal branching and included four transfers in the hand (3**b**). The digital nerve to the thumb and index finger was coapted to the recurrent motor branch of the median nerve (pink arrow), digital nerve to the index and long fingers coapted to the first lumbrical (blue arrow), digital nerve to the long and ring fingers coapted to the second lumbrical (black arrow), and digital nerve to the ring and small fingers coapted to the abductor digiti minimi (green arrow). The dorsal radial sensory branch was coapted to the branch to pronator quadratus in a separate radial-sided distal wrist incision (blue arrow, 3**c**) (color figure online)

Careful dissection of all the donor and recipient nerves at the level of amputation is important. The digital sensory nerves are identified in the radial and ulnar neurovascular bundles of each digit and traced proximally. The hypothenar, lumbrical, and interossei motor nerve branches can be identified by dissecting from distal at the muscle belly to proximal. These branches stem from the deep motor branch of the ulnar nerve. The recipient nerves should be identified using an intraoperative nerve stimulator. The dorsal radial sensory nerve is an important nerve to address as it is a source of pain if severed distally. This nerve can be coapted to the pronator quadratus (PQ) branch of AIN through a distal wrist incision radial to the flexor carpi radialis tendon to access both the donor and recipient. A case example of acute TMR in a transmetacarpal amputation is detailed in Fig. 3.

### Myoelectric considerations

Patients with shoulder, transhumeral, and transradial amputations will benefit from an ideal set of TMR coaptations to create intuitive myoelectric signals for prosthetic control. In the shoulder disarticulation patient, coaptation patterns are highly variable and conditional to the available donors and recipient nerves. Dumanian et al. defined the ideal combination as follows: The musculocutaneous nerve to the clavicular head, median nerve and ulnar nerve to split segments of the sternal head, and radial nerve to the thoracodorsal nerve [5]. Variations in this ideal pattern include using the serratus anterior and/or pectoralis minor as alternative targets (Table 1) [22, 25].

TMR in the transhumeral amputee is the most standardized combination of coaptations among the three amputation levels. Patients maintain elbow flexion and extension signals but lose hand and wrist control at this level. TMR restores these signals while protecting elbow flexion and extension for a total of five signals [5, 6, 25]. The standard pattern of transfers includes the median nerve to the short head of biceps brachii for the hand close signal, the radial nerve to the lateral head of the triceps for the hand open signal, and in cases where the residual limb is long enough, a third transfer is made with the ulnar nerve to the brachialis muscle for wrist control (Table 2) [3, 5, 6, 25–28].

Transradial amputees lose intrinsic hand function while maintaining wrist extension, flexion, supination, and pronation signals. TMR at this level requires transfer of the median and ulnar nerves to the forearm muscle to create a signal for hand closing as well as thumb abduction respectively [6, 11, 12, 29]. The median nerve is most often transferred to the FDS or PL branches as they are expendable. If unavailable, several other forearm muscles targets are available (Table 3). The ulnar nerve is nearly always transferred to the FCU branches because of its simplicity.

## Postoperative considerations

Patients are often admitted for postoperative pain management, especially in patients undergoing TMR at the time of amputation or reconstruction. Patients with delayed TMR will additionally have pain adjuncts including nerve blocks at the site of coaptation. Our preference is to place an OnQ catheter at the site of TMR to bathe the coaptations in 0.2% ropivacaine at 6 ml per hour that the patient will have in the hospital and discharged home with an OnQ ball pump, or an indwelling catheter placed by the anesthesia service.

Patients can be fitted or resume wearing their prosthesis after the soft tissue has healed, which takes 4–6 weeks. Time to reinnervation varies based on the distance between the coaptation and the neuromuscular junction with regeneration rates of 1 mm/day. Myoelectric testing and pattern recognition prosthetic fitting should be delayed until at least 3–6 months after TMR to allow for reinnervation [5, 6, 25].

The rehabilitation process is lengthy and begins prior to surgery. Success often requires a multidisciplinary approach with a team that includes at minimum a surgeon, occupational therapist, and prosthetist. Together these providers will assess the patient preoperatively for indications and likelihood of success. After surgery, rehabilitation will include exercises to activate the sensory-motor cortex including motor imagery and mirror therapy. After the nerves have reinnervated their target muscles, electrode positions and movement commands will be defined through myoelectric testing followed by training of the signals to develop control of the prosthetic. After the prosthetic is fitted and the patient learns to control it, prolonged follow up will continue to record progress and adjust the prosthetic as needed [30]. Patient factors associated with successful prosthetic rehabilitation are largely dependent on motivation and perceptions including completion of high school level education, early acceptance of the amputation, and employment at the time of amputation. Patient age and handedness were not significantly associated with successful rehabilitation [31]. Patients with less physically demanding jobs also returned to work sooner than those with physically demanding jobs [32].

Functional outcomes following TMR, although good, are limited to studies of small sample sizes, and pain outcomes have had more interest in the literature in recent years. Traditional direct control prosthetics have been the standard in amputee patients prior to the advent of TMR. However, direct control prosthetics require users to carry multiple prosthetics to perform different tasks as each prosthetic is designed for one type of function whereas myoelectric prosthetics enable users to control different functions through different myoelectric signals. Kuiken et al. demonstrated in a randomized trial of eight patients comparing direct control and myoelectric prostheses that the effects of TMR are enhanced with myoelectric (aka pattern recognition) prosthetics as demonstrated by functional tests for prosthetic users such as the Southampton Hand Assessment Procedure [33]. Several other small sample studies have demonstrated better control and greater patient satisfaction with myoelectric prosthetics following TMR [34–37].

Pain control following TMR was an unexpected benefit of the surgery and has been the focus of many recent studies of TMR in upper and lower extremity amputations. Several studies have demonstrated successful reduction of residual and phantom limb pain in delayed TMR of upper and lower extremity amputees as evidenced by improved patient reported outcomes scores and numerical ratings of pain [27, 38–42]. A randomized clinical trial in 2019 comparing traction neurectomy with TMR demonstrated patient reported outcome scores and numerical pain rating had greater improvements in the TMR group [28]. In acute TMR cases, studies have also demonstrated favorable residual and phantom limb pain scores in comparison to control groups without TMR [43, 44]. The benefits of improved amputation pain control after has become the primary indication for TMR despite its original roots in prosthetic control.

## Conclusion

Targeted muscle reinnervation was originally devised to optimize myoelectric signaling in the amputated upper limb for improved prosthetic control. Although it is a simple surgical technique, many considerations are made to provide patients with the most efficient and intuitive signals for pattern recognition prosthetic control based on the level of upper extremity amputation. Along the way, surgeons found these patients also experienced improved phantom and residual limb pain control. TMR should be considered in the upper extremity amputee, especially at an institution with the resources to accommodate a multidisciplinary team that includes a peripheral nerve surgeon, physical medicine and rehabilitation specialist, therapists, and a prosthetist.
